# Dose–response relationship of dietary Omega-3 fatty acids on slowing phenotypic age acceleration: a cross-sectional study

**DOI:** 10.3389/fnut.2024.1424156

**Published:** 2024-09-04

**Authors:** Dongzhe Wu, Yishuai Jia, Yujia Liu, Mingyu Shang

**Affiliations:** ^1^Department of Exercise Physiology, Beijing Sport University, Beijing, China; ^2^Department of Sports, China University of Geosciences, Beijing, China; ^3^Department of National Fitness, Scientific Exercise Research Center, China Institute of Sport Science, Beijing, China; ^4^Chinese Swimming Academy, Beijing Sport University, Beijing, China

**Keywords:** Omega-3, phenotypic age, phenotypic age acceleration, aging, nutritional health

## Abstract

**Purpose:**

This study investigates the association between dietary Omega-3 fatty acid intake and accelerated phenotypic aging, referred to as PhenoAgeAccel. PhenoAgeAccel is defined as the difference between phenotypic biological age, calculated using blood biochemical markers, and chronological age. This study assesses the potential of Omega-3 intake to slow biological aging and its implications for public health.

**Methods:**

Utilizing data from the NHANES from 1999 to 2018, this cross-sectional study included 20,337 adult participants. Through a nationally representative sample combined with comprehensive phenotypic age calculation methods, a cross-sectional analysis of Omega-3 fatty acid intake and accelerated phenotypic aging was conducted. Weighted generalized linear regression models and restricted cubic spline analyses were applied to explore the potential non-linear relationships between them. Threshold effects were further clarified through piecewise regression models, and the impact of different demographic and health characteristics was evaluated through interaction effect tests.

**Results:**

After adjusting for various potential confounding factors, a significant negative correlation was found between Omega-3 fatty acid intake and PhenoAgeAccel (*β* = −0.071; 95% CI: −0.119, −0.024; *p* = 0.004), indicating that an increase in Omega-3 intake is associated with a slowdown in PhenoAgeAccel. Specifically, for each unit increase in Omega-3 intake, the accelerated phenotypic aging decreased by an average of 0.071 units, revealing a significant linear negative correlation between Omega-3 intake and PhenoAgeAccel. Moreover, threshold effect analysis identified an Omega-3 fatty acid intake threshold (1.103 grams/day), beyond which the impact of Omega-3 intake on accelerated phenotypic aging tends to stabilize. Additionally, factors such as gender, age, race, and hypertension may influence the relationship between Omega-3 intake and PhenoAgeAccel, suggesting individual dietary guidance needs in different populations.

**Conclusion:**

This study highlights the potential role of dietary Omega-3 fatty acids in regulating PhenoAgeAccel and supports the strategy of delaying the aging process through dietary interventions to increase Omega-3 intake. The findings of this study contributes to the development of precise nutritional intervention strategies for different populations to optimize healthy longevity.

## 1 Introduction

Aging, as a core biological process, is complex due to a multitude of physiological and molecular changes (1). With advancing research into aging biomarkers, Phenotypic Age, as a comprehensive indicator for assessing the degree of biological aging in individuals, has garnered widespread attention. This metric quantifies the rate of aging by integrating multiple biological parameters, including but not limited to blood biomarkers, kidney function, liver function, and lung function (2). Phenotypic Age Acceleration (PhenoAgeAccel) refers to the discrepancy between an individual’s phenotypic age and their chronological age, serving as a predictor for health risks and mortality likelihood (3). For instance, research by Chen et al. (4) demonstrated that PhenoAgeAccel correlates positively with both all-cause and cause-specific mortality rates among diabetic patients, underscoring the potential value of anti-aging treatments in slowing disease progression. Additionally, Shadyab et al. (5) highlighted the potential value of PhenoAgeAccel as a biomarker in assessing cognitive impairment and brain structure, and examined whether these associations are influenced by cardiovascular diseases.

Omega-3 fatty acids, particularly DHA and EPA, play a crucial role in maintaining health. However, the endogenous production of these fatty acids in the human body is extremely limited, and blood levels of Omega-3 are primarily dependent on dietary intake (6). The American Heart Association recommends that adults consume at least 250–500 mg of DHA and EPA daily to support cardiovascular health (7). The primary dietary sources include Omega-3-rich fish such as salmon and sardines, as well as certain plant-based foods like flaxseeds and chia seeds, which contain alpha-linolenic acid (ALA). Although dietary supplements provide an alternative means of obtaining Omega-3 fatty acids, when dietary intake is sufficient, obtaining Omega-3 from natural food sources is considered more consistent with a holistic approach to healthy eating (8). Particularly, EPA and DHA, as crucial components of cell membranes, are vital for brain health and the maintenance of cognitive functions. Studies suggest that Omega-3 fatty acids, by enhancing synaptic plasticity and neurogenesis, improve cognitive functions in the elderly, exhibiting significant neuroprotective effects (9).Moreover, their anti-inflammatory properties play a crucial role in combating the chronic inflammatory states associated with aging. In terms of cardiovascular protection, Omega-3 fatty acids contribute to reducing cardiac events and lowering the risk of cardiovascular diseases, which is significant for preventing common cardiovascular issues during aging (10). Omega-3 fatty acids also positively impact maintaining a healthy metabolic rate and body composition, with studies finding Omega-3 supplementation to increase metabolic rates at rest and during exercise, and reduce body fat content, countering the common aging-associated reductions in muscle mass and metabolic slowdown (11). In cognitive function, although the findings are not consistently aligned, some studies indicate that Omega-3 supplementation helps reduce depressive symptoms in the elderly, which may indirectly reflect its positive impact on cognitive health (12). Overall, Omega-3 fatty acids have demonstrated a broad range of potential benefits in delaying aging and related diseases.

Following an in-depth analysis of the pivotal role of Omega-3 in the biological aging process, this study aims to further explore the connection between dietary Omega-3 intake and PhenoAgeAccel, and the significance of these factors in disease risk assessment and aging research, through NHANES. The study posits that these metrics hold significant potential in unveiling the subtle mechanisms of aging and in formulating effective health intervention strategies. However, despite some key discoveries, the dose–response association between dietary Omega-3 intake and PhenoAgeAccel remains not fully elucidated. Hence, our research hypothesis posits a positive correlation between high doses of dietary Omega-3 intake and a slowdown in PhenoAgeAccel, with this relationship being universal across different age groups and genders.

## 2 Methods

### 2.1 Study population

The research drew upon data from the National Health and Nutrition Examination Survey collected over the period 1999–2018. This survey, a project of the Centers for Disease Control and Prevention, is designed to evaluate the health and nutritional status of the civilian, non-institutionalized population of the United States. Orchestrated by the CDC National Center for Health Statistics, NHANES administers a stratified multistage probability sampling strategy to survey roughly 10,000 Americans biennially. The data acquisition process encompasses in-depth interviews conducted within households and thorough physical assessments. The interview process elicits detailed demographic, socioeconomic, dietary, and health-related information. The physical assessments comprise comprehensive evaluations of medical, dental, and a range of physiological-biochemical markers.

**Figure 1:**
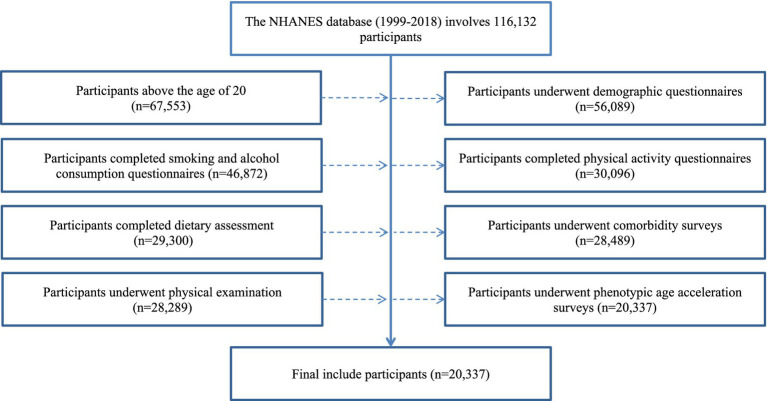
Flow chart.

In this investigation, a rigorous selection protocol was employed, ultimately including 20,337 adults for analysis. This protocol predominantly involved eliminating subjects who did not conform to the age requirements and those who were unable to complete the necessary surveys and physical assessments. Data were analyzed using a complete case analysis strategy. The intricate details of the selection process are illustrated in Figure 1. The NHANES survey data utilized in this research received appropriate ethical clearance, conforming to the ethical standards set forth in the Declaration of Helsinki.

### 2.2 Definitions of phenotypic age acceleration

This study builds upon prior significant research on phenotypic age, employing an integrative approach to compute an individual’s phenotypic age (3). The algorithm utilizes clinical laboratory blood chemistry parameters, incorporating multiple biomarkers such as albumin, creatinine, glucose, total white blood cell count, percentage of lymphocytes, red cell distribution width, mean corpuscular volume, and alkaline phosphatase. These biomarkers were modeled using elastic-net regression within NHANES III. Due to the absence of C-reactive protein (CRP) data from 2011 to 2018, CRP was not included as a clinical biomarker in our study, following precedents from previous research (13). Notably, earlier studies comparing phenotypic ages calculated with and without CRP biomarker sets found a high correlation between the two (correlation coefficient of 0.99) (14, 15), indicating that the absence of CRP has a limited impact on the calculation of phenotypic age.

Particular attention was given to PhenoAgeAccel, a metric derived from the residuals of a linear regression analysis between phenotypic age and chronological age (16, 17). For instance, if two individuals are of the same age but one exhibits a younger state due to better physiological health and vitality, while the other appears older due to health issues or poor lifestyle choices, their PhenoAgeAccel values will differ. Serving as a de-merit indicator, lower values of PhenoAgeAccel represent a slower biological aging process, providing a crucial perspective on the discrepancy between an individual’s physiological state and their actual age.

The specific formula is as follows:


$$\begin{array}{c} \mathrm{PhenoAge}=143.5671\\ +\frac{\ln\left[-0.0059383581\times \ln\left[1-\mathrm{mortality risk}\right]\right]}{0.08548908}\\ \;\mathrm{mortality risk}=1-e^{-e^{{\mathrm{xb}}_{\left[\exp\left(120x\gamma \right)-1\right]/\gamma }}}\\ \;\gamma =0.007354285 \end{array}$$

$$\begin{array}{c} xb=-18.311623487-0.029197296\times \mathrm{albumin}\\ +0.002285539\times \mathrm{alkaline}\;\mathrm{phosphata}\\ +0.006379140\times \mathrm{creatinine}\\ +0.177752648\\ \times \mathrm{glycated}\;\mathrm{hemoglobin}\\ +0.055248172\\ \times \mathrm{white blood cell count}\\ -0.013502137\\ \times \mathrm{lymphocyte percentage}\\ +0.029331315\\ \times \mathrm{mean corpuscular volume}\\ +0.234452108\\ \times \mathrm{red}\;\mathrm{cell distribution width}\\ +0.077245523\times \mathrm{chronological}\;\mathrm{age} \end{array}$$

### 2.3 Definitions of Omega-3

This research estimated total omega-3 fatty acid intake by totaling the consumed amounts of alpha-linolenic acid (ALA, 18:3n-3), docosapentaenoic acid (DPA, 22:5n-3), eicosatetraenoic acid (ETA, 20:4n-3), eicosapentaenoic acid (EPA, 20:5n-3), and docosahexaenoic acid (DHA, 22:6n-3) (18). Data on omega-3 intake was sourced from the 24-h dietary recall and the Food Frequency Questionnaire (FFQ), employing the Automated Multiple-Pass Method (AMPM) to ensure a maximum deviation of 10% from actual intake (19) (20). Initial dietary data was collected from participants by trained interviewers through detailed interviews, with a follow-up via telephone occurring 3–10 days later to confirm consistency. Omega-3 intake calculations were derived from the mean of two 24-h dietary recalls, or from the initial recall if only one was completed.

### 2.4 Covariate

This study incorporated multiple covariates for a comprehensive analysis, including basic demographic information, lifestyle factors, and health status indicators. The basic demographic information encompassed age, gender (male, female), ethnic backgrounds were categorized as Mexican American, Non-Hispanic Black, Non-Hispanic White, among others, and levels of education were delineated as below high school, at high school level, and above high school education.Economic classification was gaged through the Poverty Income Ratio (PIR), a scale juxtaposing the income of a household or individual against the annual poverty benchmarks (categorized as low income for a PIR of 1.3 or less, moderate income for a PIR >1.3 but <3.5, and high income for a PIR of 3.5 or above).

Lifestyle factors included marital status (married/cohabiting, widowed/divorced/separated), body mass index (BMI) categories (<25, 25–29.9, ≥30 kg/m^2^), smoking status (never, former, current), and alcohol status (never, former, light, moderate, heavy). Physical activity, in the preceding week, the quantification of physical activity, encompassing both vigorous and moderate exertions undertaken during occupational, commuting, and leisure intervals, was operationalized via the metric of metabolic equivalent tasks, expressed in weekly MET-hours. Dietary quality was assessed by the Healthy Eating Index 2015 (HEI-2015), which evaluates the consistency of an individual’s or group’s diet with the Dietary Guidelines for Americans published by the United States Department of Agriculture (21). This index comprises several components, each representing a critical aspect of the diet, such as fruits, vegetables, whole grains, dairy, proteins, the ratio of unsaturated to saturated fats, sodium, and added sugars intake. The HEI-2015 scoring system is quantitative, with a total score of 100, where higher scores indicate better diet quality and greater adherence to the Dietary Guidelines for Americans (22).

Health status indicators included criteria for diagnosing hypertension, hyperlipidemia, and diabetes. Hypertension was determined based on physician diagnosis, use of antihypertensive medication, and blood pressure measurements (systolic ≥140 mmHg or diastolic ≥90 mmHg). The diagnosis of hyperlipidemia was based on lipid levels (triglycerides, total cholesterol, LDL, and HDL levels) and the use of lipid-lowering medications. Diabetes criteria were based on physician diagnosis, glycated hemoglobin levels, fasting glucose levels, and the use of antidiabetic medications. Cancer prevalence was determined by diagnosis from a physician or health professional.

### 2.5 Statistical analysis

In the investigation, rigorous adherence to the detailed sampling methodologies prescribed by the NHANES was maintained, with the computation of complex sampling weights conforming to NHANES analytical standards. To reflect the U.S. population accurately, weighted data underpinned all statistical evaluations. Continuous measures were expressed as means with their respective standard errors (Mean ± SE), and categorical data were depicted through frequency counts and their corresponding weighted proportions.

For discerning group variances, the analysis applied one-way analysis of variance for continuous measures and Chi-square testing for categorical data. In addition, this research incorporated weighted generalized linear regression models to probe the association between dietary omega-3 fatty acid consumption and PhenoAgeAccel, with further subgroup analyses by sex to examine potential gender-based effects on this linkage.

Additionally, based on the outcomes of the linear regression model, this research employed restricted cubic splines to examine the non-linear trends between variables. For associations exhibiting non-linear trends, the study further elucidated the threshold effects of dietary omega-3 intake and PhenoAgeAccel through piecewise regression combined with likelihood ratio tests. Lastly, to validate the impact of control variables on the relationship between dietary Omega-3 intake and PhenoAgeAccel, these variables were incorporated into the interaction effect test model.

All statistical analyses were conducted with a two-tailed *p* < 0.05 as the threshold for statistical significance, using R Studio (version 4.2.1, United States) for data processing and analysis.

## 3 Results

### 3.1 Baseline characteristics of the participants

This study encompassed baseline characteristics of 20,337 adults, stratified into four quartiles based on PhenoAgeAccel. Within these quartiles, significant differences in Omega-3 intake were observed, demonstrating fluctuating variations across different quartiles with PhenoAgeAccel increase, without a consistent trend.

Significant differences in the distribution of sex, age, and ethnicity were also evident across different PhenoAgeAccel quartiles. Females constituted the highest proportion in the first quartile (72.27%), whereas males were predominant in the fourth quartile (69.47%). The age group distribution indicated that participants aged 60 and above increased in proportion with rising PhenoAgeAccel. Ethnic distribution revealed that Non-Hispanic White formed the majority in all quartiles.

The distribution of BMI indicated an increase in the proportion of individuals with BMI ≥ 30 as PhenoAgeAccel increased. Regarding marital status, the proportion of married or cohabiting individuals decreased with higher PhenoAgeAccel. The distribution of educational levels showed a decrease in the proportion of individuals with higher education as PhenoAgeAccel increased. The poverty income ratio (PIR) displayed that lower-income groups constituted a larger proportion in higher PhenoAgeAccel quartiles.

Lifestyle factors such as smoking and alcohol consumption status also exhibited significant differences across PhenoAgeAccel quartiles. Physical activity levels were significantly higher in the first quartile compared to other groups. The Healthy Eating Index indicated a decline in diet quality with an increase in PhenoAgeAccel.

In terms of chronic disease conditions, the distribution of diabetes and hypertension showed significant differences across different PhenoAgeAccel quartiles, with participants without these conditions being more prevalent in lower PhenoAgeAccel quartiles. The distribution of hyperlipidemia did not show significant differences across quartiles, whereas the prevalence of cancer was slightly higher in higher PhenoAgeAccel quartiles (Table 1).

**Table 1:** Baseline characteristics of the participants.

Characteristic	Overall	Quantile 1 [−18.38, −5.65]	Quantile 2 (−5.65, −2.83]	Quantile 3 (−2.83, 0.13]	Quantile 4 (0.13, 23.38]	*P*-value
N	20,337	5,084	5,084	5,082	5,087	
PhenoAgeAccel	−2.90 (0.07)	−8.04 (0.04)	−4.19 (0.01)	−1.42 (0.02)	3.02 (0.05)	<0.0001
Omega-3, gram/day	1.84 (0.02)	1.82 (0.03)	1.83 (0.02)	1.90 (0.02)	1.82 (0.03)	0.02
Gender, *n* (weighted %)						< 0.0001
Female	9,441 (48.09)	3,634 (72.27)	2,504 (50.55)	1815 (35.72)	1,488 (30.53)	
Male	10,896 (51.91)	1,450 (27.73)	2,580 (49.45)	3,267 (64.28)	3,599 (69.47)	
Age, years, *n* (weighted %)						< 0.0001
20–29	3,393 (18.11)	699 (13.68)	923 (19.27)	927 (20.08)	844 (19.74)	
30–39	3,752 (20.42)	964 (20.30)	955 (20.19)	991 (21.70)	842 (19.38)	
40–49	3,812 (21.83)	1,073 (23.61)	969 (22.36)	877 (20.14)	893 (20.98)	
50–59	3,249 (18.99)	914 (21.74)	797 (18.61)	755 (17.74)	783 (17.59)	
≥60	6,131 (20.66)	1,434 (20.67)	1,440 (19.57)	1,532 (20.34)	1725 (22.30)	
Race, *n* (weighted %)						< 0.0001
Non-Hispanic White	10,538 (74.53)	2,510 (74.47)	2,753 (76.58)	2,719 (75.15)	2,556 (71.48)	
Mexican American	3,429 (6.64)	951 (6.76)	856 (6.38)	848 (6.67)	774 (6.79)	
Non-Hispanic Black	3,564 (8.67)	752 (7.16)	766 (7.21)	904 (8.81)	1,142 (12.05)	
Other race (including multi-racial and other Hispanic)	2,806 (10.15)	871 (11.61)	709 (9.83)	611 (9.37)	615 (9.68)	
BMI, kg/m^2^, *n* (weighted %)						< 0.0001
<25	6,075 (32.06)	2,202 (48.03)	1,687 (35.16)	1,256 (24.62)	930 (17.90)	
25–29.9	6,999 (33.43)	1800 (33.09)	1835 (35.52)	1805 (35.47)	1,559 (29.06)	
≥30	7,263 (34.51)	1,082 (18.88)	1,562 (29.32)	2021 (39.91)	2,598 (53.04)	
Marital status, *n* (weighted %)						< 0.0001
Married/living with partner	13,033 (67.80)	3,422 (72.40)	3,326 (69.23)	3,207 (66.36)	3,078 (62.29)	
Never married	3,319 (16.17)	659 (11.81)	847 (16.14)	910 (18.04)	903 (19.27)	
Widowed/divorced/separated	3,985 (16.02)	1,003 (15.79)	911 (14.63)	965 (15.59)	1,106 (18.44)	
Education, *n* (weighted %)						< 0.0001
Below	4,308 (12.59)	1,003 (11.07)	976 (11.00)	1,092 (12.84)	1,237 (16.00)	
High school	4,785 (24.03)	991 (18.35)	1,113 (22.60)	1,287 (26.79)	1,394 (29.35)	
Above	11,244 (63.38)	3,090 (70.59)	2,995 (66.39)	2,703 (60.37)	2,456 (54.65)	
PIR, *n* (weighted %)						< 0.0001
<1.3	5,064 (16.34)	1,115 (12.97)	1,139 (14.54)	1,288 (17.27)	1,522 (21.40)	
1.3–3.49	7,754 (34.53)	1886 (32.85)	1907 (33.69)	1938 (34.96)	2023 (37.00)	
≥3.5	7,519 (49.14)	2083 (54.18)	2038 (51.77)	1856 (47.76)	1,542 (41.59)	
Smoke status, *n* (weighted %)						< 0.0001
Former smoker	5,330 (25.51)	1,248 (25.14)	1,294 (25.42)	1,432 (26.86)	1,356 (24.54)	
Nonsmoker	10,621 (52.89)	3,214 (62.55)	2,853 (56.00)	2,439 (48.46)	2,115 (42.75)	
Current smoker	4,386 (21.61)	622 (12.31)	937 (18.59)	1,211 (24.68)	1,616 (32.71)	
Alcohol status, *n* (weighted %)						< 0.0001
Former	3,095 (12.41)	698 (11.31)	687 (11.25)	828 (12.94)	882 (14.50)	
Never	2,294 (9.04)	738 (11.57)	577 (8.42)	504 (8.23)	475 (7.71)	
Mild	7,317 (38.20)	1906 (40.15)	1919 (40.77)	1841 (37.50)	1,651 (33.65)	
Moderate	3,352 (18.47)	989 (22.20)	842 (18.60)	793 (17.12)	728 (15.44)	
Heavy	4,279 (21.87)	753 (14.78)	1,059 (20.96)	1,116 (24.21)	1,351 (28.69)	
Physical activity, MET-min/wk., *n* (weighted %)						0.004
Q1 [0.93,504]	6,819 (33.18)	1,647 (32.02)	1724 (32.77)	1,672 (33.15)	1776 (35.06)	
Q2 (504, 2,160]	6,787 (34.35)	1863 (37.32)	1,685 (34.85)	1,658 (33.38)	1,581 (31.36)	
Q3 (2,160,58,320]	6,731 (32.47)	1,574 (30.66)	1,675 (32.38)	1752 (33.47)	1730 (33.58)	
Healthy eating index, *n* (weighted %)						< 0.0001
Q1 [0,44.27]	6,779 (34.84)	1,315 (25.77)	1,636 (33.58)	1787 (37.46)	2041 (44.07)	
Q2 (44.27,56.38]	6,779 (33.15)	1,648 (32.70)	1,660 (32.36)	1736 (33.98)	1735 (33.69)	
Q3 (56.38,96.35]	6,779 (32.01)	2,121 (41.53)	1788 (34.06)	1,559 (28.56)	1,311 (22.24)	
Diabetes, *n* (weighted %)						< 0.0001
No	16,126 (83.57)	4,463 (90.21)	4,336 (88.77)	4,044 (83.00)	3,283 (70.19)	
Yes	4,211 (16.43)	621 (9.79)	748 (11.23)	1,038 (17.00)	1804 (29.81)	
Hypertension, *n* (weighted %)						< 0.0001
No	12,378 (66.26)	3,811 (78.19)	3,415 (71.33)	2,937 (63.08)	2,215 (49.74)	
Yes	7,959 (33.74)	1,273 (21.81)	1,669 (28.67)	2,145 (36.92)	2,872 (50.26)	
Hyperlipidemia, *n* (weighted %)						< 0.0001
No	5,756 (29.58)	1,589 (32.41)	1,502 (31.01)	1,416 (29.04)	1,249 (25.16)	
Yes	14,581 (70.42)	3,495 (67.59)	3,582 (68.99)	3,666 (70.96)	3,838 (74.84)	
Cancer, *n* (weighted %)						0.03
No	18,536 (91.36)	4,635 (90.30)	4,677 (92.16)	4,615 (91.79)	4,609 (91.19)	
Yes	1801 (8.64)	449 (9.70)	407 (7.84)	467 (8.21)	478 (8.81)	

Continuous variables are summarized as mean values with standard errors, and categorical variables are presented as counts with their weighted percentages; One-way ANOVA was utilized for continuous data, while the Chi-square test was employed for categorical data. Quantile 1, Q1—first quartile; Quantile 2, Q2—second quartile; Quantile 3, Q3—third quartile; Quantile 4, Q4—fourth quartile.

### 3.2 Association analysis between Omega-3 intake and phenoptypic age acceleration in American adults

**Figure 2:**
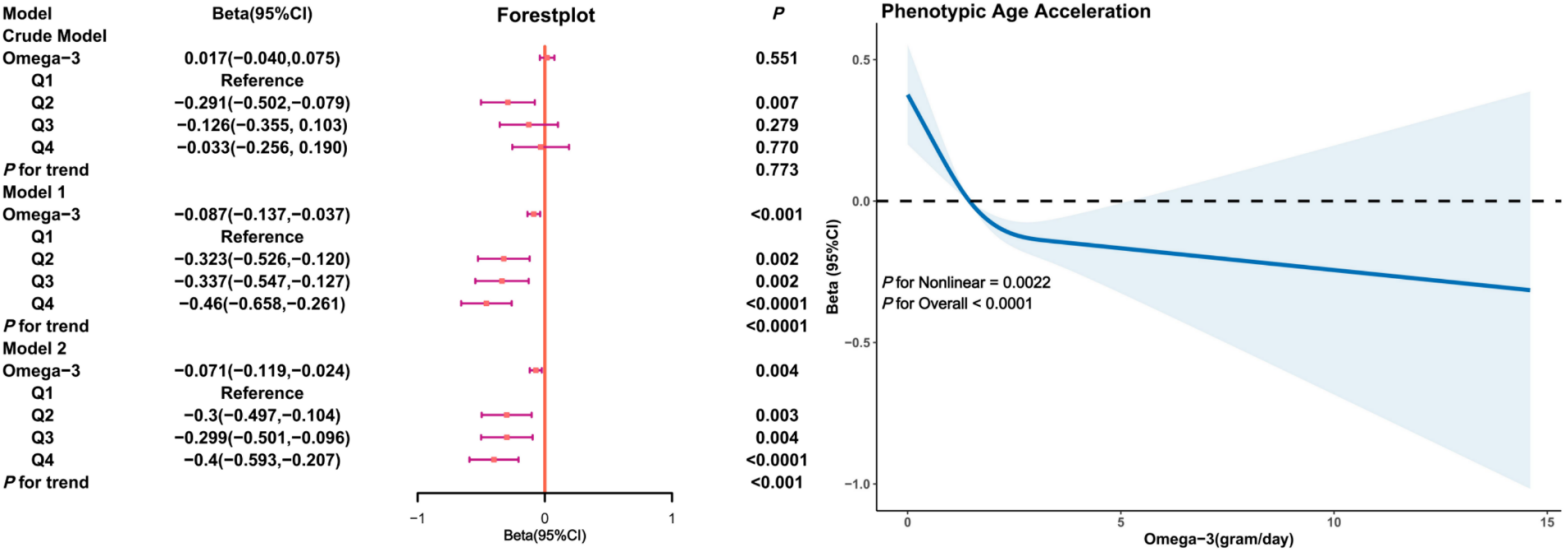
Association analysis between Omega-3 intake and phenoptypic age acceleration in American adults. Crude Model is the unadjusted model. Model 1 adjusted for age, gender, race, PIR, education, BMI, marital status, smoke, alcohol, physical activity, HEI. Model 2 adjusted for age, gender, race, PIR, education, BMI, marital status, smoke, alcohol, physical activity, HEI, diabetes, hypertension, hyperlipidemia, and cancer.

Figure 2 illustrates the relationship between Omega-3 intake and PhenoAgeAccel in unadjusted (crude model) and adjusted models. In the crude model, the association between Omega-3 intake and PhenoAgeAccel was not significant (*β* = 0.017, 95% CI: −0.040, 0.075, *p* = 0.551). However, after adjusting for age, gender, race, PIR, education level, BMI, marital status, smoking and drinking status, physical activity, and HEI (Model 1), and further adjusting for diabetes, hypertension, hyperlipidemia, and cancer (Model 2), a significant negative correlation between Omega-3 intake and PhenoAgeAccel was observed (*β* = −0.46 in Q4 of Model 1; *β* = −0.40 in Q4 of Model 2). Restricted cubic splines indicated a non-linear trend between Omega-3 intake and PhenoAgeAccel (*P* for Nonlinear = 0.0022), suggesting that as Omega-3 intake increases, PhenoAgeAccel tends to decrease with a threshold effect evident.

**Figure 3:**
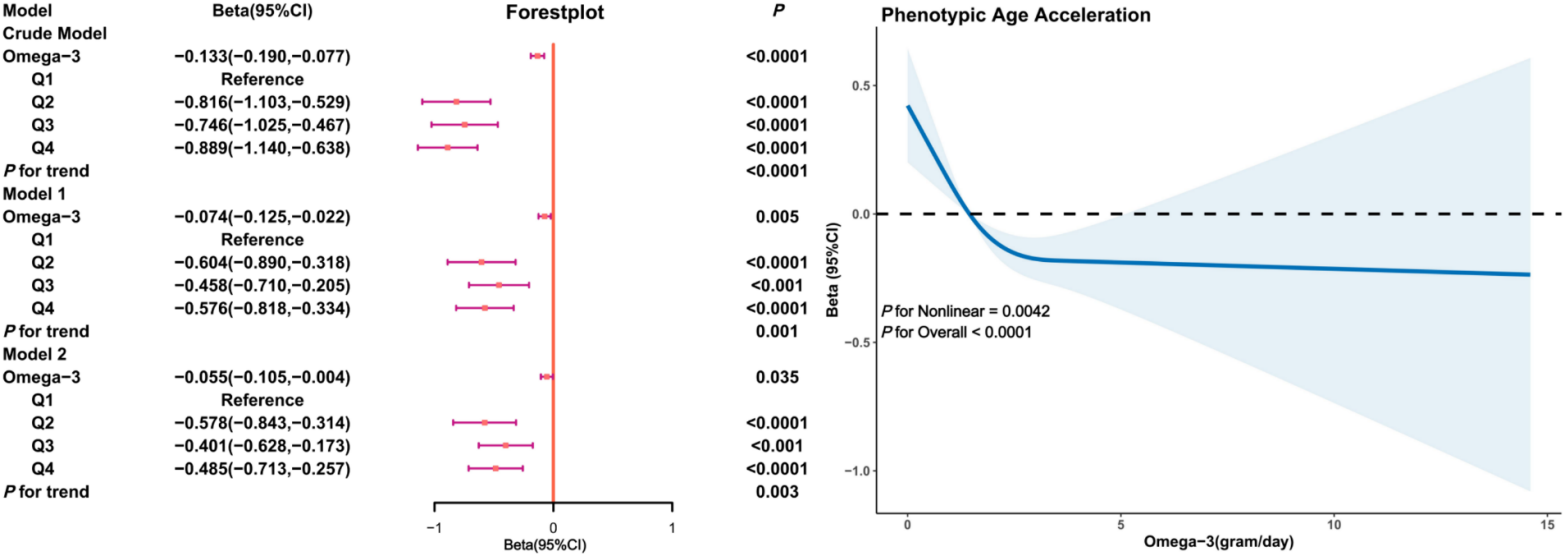
Association analysis between Omega-3 intake and phenoptypic age acceleration in American adults (gender subgroup: male). Crude Model is the unadjusted model. Model 1 adjusted for age, race, PIR, education, BMI, marital status, smoke, alcohol, physical activity, HEI. Model 2 adjusted for age, race, PIR, education, BMI, marital status, smoke, alcohol, physical activity, HEI, diabetes, hypertension, hyperlipidemia, and cancer.

In the male subgroup (Figure 3), a significant negative correlation between Omega-3 intake and PhenoAgeAccel was present in both the crude and adjusted models. In Model 2, the Beta coefficient for Omega-3 intake in the fourth quartile was (*β* = −0.485, 95% CI: −0.713, −0.257, *p* < 0.0001) with restricted cubic splines indicating a non-linear trend between Omega-3 intake and PhenoAgeAccel in males (*P* for Nonlinear = 0.0042), showing a significant decline in PhenoAgeAccel with increased Omega-3 intake and a threshold effect.

**Figure 4:**
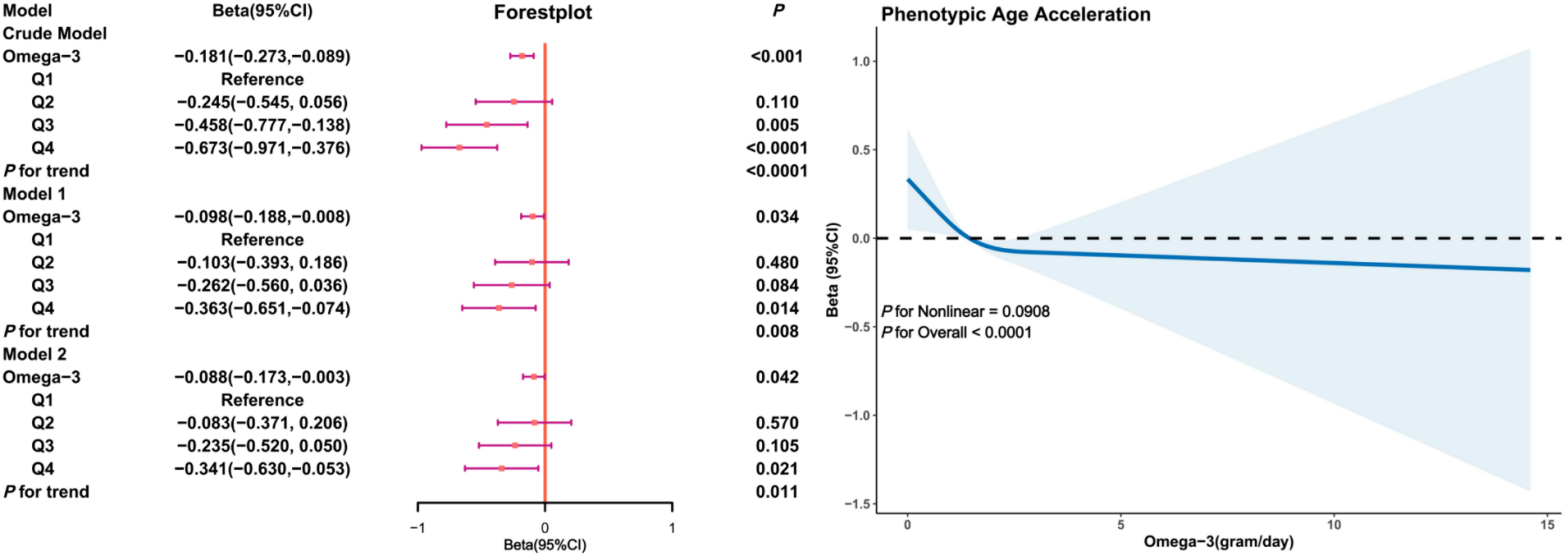
Association analysis between Omega-3 intake and phenoptypic age acceleration in American adults (gender subgroup: female). Crude Model is the unadjusted model. Model 1 adjusted for age, race, PIR, education, BMI, marital status, smoke, alcohol, physical activity, HEI. Model 2 adjusted for age, race, PIR, education, BMI, marital status, smoke, alcohol, physical activity, HEI, diabetes, hypertension, hyperlipidemia, and cancer.

In the female subgroup (Figure 4), a negative correlation between Omega-3 intake and PhenoAgeAccel was also observed, particularly in the adjusted models. Model 2 revealed that, compared to the first quartile, the Beta coefficient for Omega-3 intake in the fourth quartile was (*β* = −0.341, 95% CI: −0.630, −0.053, *p* = 0.011) with restricted cubic splines indicating a non-linear trend between Omega-3 intake and PhenoAgeAccel in females (*P* for Nonlinear = 0.0908), implying that in the female subgroup, an increase in Omega-3 intake is associated with a decline in PhenoAgeAccel without a threshold effect.

### 3.3 Threshold effect analysis of the relationship between Omega-3 intake and Phenoptypic age acceleration

This study examined the relationship between Omega-3 intake and PhenoAgeAccel in U.S. adults, with a particular focus on the presence of a threshold effect of Omega-3 intake. In the full model, a linear regression analysis revealed a significant linear negative association between Omega-3 intake and PhenoAgeAccel (*β* = −0.071, 95% CI: −0.119, −0.024, *p* = 0.004). Further analysis using a two-piece segmented linear regression model identified a significant inflection point at an Omega-3 intake of 1.103 grams/day, where the relationship between intake and PhenoAgeAccel changed. Below this inflection point, the intake was significantly negatively associated with PhenoAgeAccel (*β* = −0.482, 95% CI: −0.716, −0.248, *p* = 0.0001), whereas beyond this point, the relationship was no longer significant (*β* = −0.041, 95% CI: −0.086, 0.004, *p* = 0.0753). The log-likelihood ratio test supported the significant superiority of the segmented linear model over the single linear model (*p* < 0.001).

In the gender-stratified analysis, the male model also indicated that the two-piece segmented linear regression model was more appropriate than a single linear regression, with the inflection point set at an Omega-3 intake of 0.461 grams/day. Below this inflection point, the significant negative correlation between Omega-3 intake and PhenoAgeAccel was stronger (*β* = −2.921, 95% CI: −4.688, −1.155, *p* = 0.0012), while above the inflection point, this negative correlation weakened but remained significant (*β* = −0.064, 95% CI: −0.113, −0.015, *p* = 0.011) (Table 2).

**Table 2:** Threshold effect analysis of the relationship between Omega-3 intake and phenoptypic age acceleration.

Outcome	*β*	95% CI	*P*-value
All model			
One-line linear regression model	−0.071	−0.119, −0.024	0.004
Two-piecewise linear regression model			
Inflection point	1.103		
Omega-3 intake < 1.103 (gram/day)	−0.482	−0.716, −0.248	0.0001
Omega-3 intake ≥ 1.103 (gram/day)	−0.041	−0.086,0.004	0.0753
*P* for log-likelihood ratio test	<0.001		<0.001
Male Model			
One-line linear regression model	−0.055	−0.105, −0.004	0.035
Two-piecewise linear regression model			
Inflection point	0.461		
Omega-3 intake < 0.461 (gram/day)	−2.921	−4.688, −1.155	0.0012
Omega-3 intake ≥ 0.461 (gram/day)	−0.064	−0.113, −0.015	0.011
*P* for log-likelihood ratio test			0.002

Adjusted for age, gender, race, PIR, education, BMI, marital status, smoke, alcohol, physical activity, HEI, diabetes, hypertension, hyperlipidemia, and cancer.

### 3.4 Interaction effect test

In this study, the relationship between Omega-3 intake and PhenoAgeAccel was explored for potential interactions with demographic and health-related characteristics such as gender, age, ethnicity, education level, economic status, BMI, marital status, smoking and drinking habits, physical activity, diet quality, and chronic disease conditions through interaction effect tests. The results indicated that there were interactive effects between Omega-3 intake and factors such as gender, age, and ethnicity. The analysis suggested that females might derive greater benefits from Omega-3 intake in slowing down PhenoAgeAccel, with individuals aged 60 and above showing a more pronounced negative effect. Ethnic differences also influenced the efficacy of Omega-3, particularly among Non-Hispanic White and Non-Hispanic Black. Moreover, the presence of hypertension showed a significant interactive effect with the relationship between Omega-3 intake and PhenoAgeAccel. However, other factors such as education, PIR, BMI, marital status, smoking and drinking habits, physical activity, and diet quality did not exhibit significant interactions. These findings reveal individual variability in the impact of Omega-3 intake on biological aging across different populations, emphasizing the need for future research to consider individuals’ biological and sociodemographic characteristics to optimize nutritional recommendations (Table 3).

**Table 3:** Interaction effect test (Omega-3).

Characteristic	Q1	Q2	*P*	Q3	*P*	Q4	*P*	*P* for trend	*P* for interaction
Gender									0.03
Female	ref	−0.58 (−0.84, −0.31)	<0.0001	−0.4 (−0.63, −0.17)	<0.001	−0.49 (−0.71, −0.26)	<0.0001	<0.001	
Male	ref	−0.08 (−0.37, 0.21)	0.57	−0.23 (−0.52, 0.05)	0.10	−0.34 (−0.63, −0.05)	0.02	0.1	
Age									0.02
20–29	ref	−0.21 (−0.60, 0.18)	0.29	−0.27 (−0.61, 0.06)	0.11	−0.43 (−0.82, −0.04)	0.03	0.46	
30–39	ref	−0.39 (−0.74, −0.03)	0.03	−0.28 (−0.67, 0.11)	0.16	−0.49 (−0.92, −0.06)	0.03	0.18	
40–49	ref	−0.21 (−0.65, 0.24)	0.36	0.1 (−0.33, 0.52)	0.66	0.17 (−0.25, 0.58)	0.42	0.13	
50–59	ref	−0.15 (−0.60, 0.30)	0.50	−0.03 (−0.47, 0.42)	0.91	−0.06 (−0.50, 0.38)	0.78	0.97	
≥60	ref	−0.52 (−0.88, −0.17)	0.004	−0.9 (−1.32, −0.49)	<0.0001	−0.96 (−1.36, −0.56)	<0.0001	<0.001	
Race									0.01
Non-Hispanic White	ref	−0.35 (−0.59, −0.11)	0.01	−0.39 (−0.65, −0.13)	0.003	−0.46 (−0.71, −0.22)	<0.001	0.96	
Mexican American	ref	−0.34 (−0.72, 0.03)	0.07	−0.25 (−0.68, 0.17)	0.24	−0.41 (−0.83, 0.01)	0.05	0.33	
Non-Hispanic Black	ref	−0.24 (−0.63, 0.16)	0.23	−0.38 (−0.80, 0.03)	0.07	−0.56 (−0.97, −0.14)	0.01	0.25	
Other Race	ref	0.02 (−0.44, 0.48)	0.95	0.49 (0.03, 0.96)	0.04	0.31 (−0.15, 0.77)	0.18	0.58	
Education									0.56
Below	ref	−0.59 (−0.98, −0.19)	0.005	−0.4 (−0.82, 0.02)	0.06	−0.23 (−0.69, 0.23)	0.32	0.004	
High School	ref	−0.25 (−0.68, 0.17)	0.24	−0.28 (−0.66, 0.11)	0.16	−0.61 (−0.97, −0.25)	0.001	0.25	
Above	ref	−0.26 (−0.51, −0.01)	0.04	−0.29 (−0.52, −0.06)	0.02	−0.34 (−0.57, −0.11)	0.004	0.06	
PIR									0.11
<1.3	ref	−0.13 (−0.48, 0.23)	0.48	−0.23 (−0.57, 0.12)	0.19	−0.24 (−0.57, 0.09)	0.15	0.46	
1.3–3.49	ref	−0.47 (−0.79, −0.15)	0.004	−0.21 (−0.50, 0.07)	0.15	−0.66 (−0.96, −0.36)	<0.0001	<0.0001	
≥3.5	ref	−0.22 (−0.52, 0.07)	0.14	−0.35 (−0.67, −0.04)	0.03	−0.24 (−0.52, 0.05)	0.10	0.49	
BMI									0.82
<25	ref	−0.24 (−0.57, 0.08)	0.14	−0.28 (−0.61, 0.05)	0.10	−0.35 (−0.66, −0.04)	0.03	0.17	
25–29.9	ref	−0.48 (−0.77, −0.18)	0.002	−0.33 (−0.60, −0.06)	0.02	−0.53 (−0.83, −0.24)	<0.001	0.03	
≥30	ref	−0.21 (−0.52, 0.11)	0.19	−0.33 (−0.67, 0.01)	0.06	−0.33 (−0.67, 0.00)	0.05	0.27	
Marital status									0.43
Widowed/divorced/separated	ref	−0.25 (−0.47, −0.03)	0.03	−0.28 (−0.52, −0.04)	0.02	−0.35 (−0.57, −0.13)	0.002	0.1	
Never married	ref	−0.33 (−0.76, 0.10)	0.13	−0.55 (−0.98, −0.12)	0.01	−0.4 (−0.85, 0.06)	0.09	0.17	
Married/living with partner	ref	−0.42 (−0.86, 0.03)	0.06	−0.09 (−0.50, 0.33)	0.68	−0.57 (−0.95, −0.18)	0.005	0.002	
Smoke status									0.85
Former smoker	ref	−0.41 (−0.81, 0.00)	0.05	−0.34 (−0.75, 0.06)	0.10	−0.49 (−0.91, −0.07)	0.02	0.05	
Nonsmoker	ref	−0.35 (−0.71, 0.02)	0.06	−0.19 (−0.56, 0.17)	0.30	−0.35 (−0.73, 0.03)	0.07	0.14	
Current smoker	ref	−0.22 (−0.48, 0.03)	0.08	−0.32 (−0.57, −0.07)	0.01	−0.38 (−0.64, −0.12)	0.005	0.02	
Alcohol status									0.16
Former	ref	−0.49 (−1.03, 0.04)	0.07	−0.37 (−0.93, 0.18)	0.19	−0.73 (−1.22, −0.24)	0.004	0.04	
Never	ref	−0.19 (−0.74, 0.36)	0.50	−0.7 (−1.25, −0.14)	0.01	−0.11 (−0.69, 0.47)	0.70	0.2	
Mild	ref	−0.41 (−0.67, −0.15)	0.002	−0.33 (−0.63, −0.03)	0.03	−0.37 (−0.61, −0.12)	0.004	0.06	
Moderate	ref	0.14 (−0.27, 0.54)	0.50	−0.11 (−0.51, 0.29)	0.58	−0.16 (−0.57, 0.25)	0.44	0.16	
Heavy	ref	−0.42 (−0.84, −0.01)	0.04	−0.25 (−0.64, 0.14)	0.20	−0.61 (−1.02, −0.20)	0.004	0.2	
Physical activity									0.87
Q1	ref	−0.26 (−0.55, 0.03)	0.08	−0.24 (−0.59, 0.10)	0.16	−0.41 (−0.72, −0.10)	0.01	0.53	
Q2	ref	−0.24 (−0.56, 0.08)	0.14	−0.31 (−0.68, 0.07)	0.10	−0.48 (−0.81, −0.15)	0.005	0.03	
Q3	ref	−0.44 (−0.81, −0.07)	0.02	−0.37 (−0.68, −0.06)	0.02	−0.32 (−0.66, 0.01)	0.06	0.65	
Healthy eating index									0.1
Q1	ref	−0.3 (−0.59, 0.00)	0.05	−0.16 (−0.46, 0.14)	0.30	−0.35 (−0.64, −0.07)	0.01	0.02	
Q2	ref	−0.28 (−0.62, 0.06)	0.10	−0.09 (−0.43, 0.24)	0.58	−0.4 (−0.75, −0.05)	0.02	0.54	
Q3	ref	−0.32 (−0.66, 0.01)	0.06	−0.68 (−1.00, −0.35)	<0.0001	−0.49 (−0.82, −0.16)	0.004	0.03	
Diabetes									0.72
Yes	ref	−0.3 (−0.51, −0.10)	0.004	−0.34 (−0.54, −0.13)	0.002	−0.39 (−0.59, −0.20)	<0.001	0.18	
No	ref	−0.26 (−0.75, 0.23)	0.30	−0.08 (−0.66, 0.50)	0.78	−0.42 (−0.94, 0.10)	0.11	0.52	
Hyperlipidemia									0.19
Yes	ref	−0.38 (−0.61, −0.14)	0.002	−0.38 (−0.61, −0.15)	0.002	−0.53 (−0.74, −0.31)	<0.0001	0.22	
No	ref	−0.11 (−0.45, 0.22)	0.50	−0.08 (−0.44, 0.27)	0.63	−0.08 (−0.39, 0.23)	0.61	0.53	
Hypertension									0.002
Yes	ref	−0.66 (−0.97, −0.35)	<0.0001	−0.45 (−0.80, −0.11)	0.01	−0.81 (−1.11, −0.51)	<0.0001	0.93	
No	ref	−0.11 (−0.36, 0.14)	0.37	−0.21 (−0.44, 0.02)	0.07	−0.18 (−0.42, 0.06)	0.15	0.26	
Cancer									0.39
Yes	ref	−0.27 (−0.47, −0.07)	0.01	−0.28 (−0.48, −0.07)	0.01	−0.42 (−0.62, −0.21)	<0.0001	0.26	
No	ref	−0.72 (−1.35, −0.08)	0.03	−0.62 (−1.29, 0.04)	0.07	−0.27 (−0.86, 0.32)	0.37	0.37	

## 4 Discussion

This study explored the relationship between dietary Omega-3 intake and PhenoAgeAccel, finding a significant negative correlation between Omega-3 intake and PhenoAgeAccel after adjusting for multiple potential confounding factors. This finding supports the hypothesis that Omega-3 fatty acids may play a positive role in slowing the aging process and suggests that increasing Omega-3 intake could have potential benefits in delaying biological aging. Notably, this study revealed a nonlinear relationship between Omega-3 intake and PhenoAgeAccel, indicating a threshold of intake (1.103 grams/day), beyond which the negative correlation between Omega-3 intake and PhenoAgeAccel weakens. This finding suggests that there may be an optimal range for Omega-3 intake, with no additional anti-aging benefits beyond this range. Moreover, the study indicates that factors such as gender, age, ethnicity, and hypertension may influence the relationship between Omega-3 intake and PhenoAgeAccel. For instance, males and participants aged 60 and above exhibited a stronger negative correlation with Omega-3 intake, possibly reflecting differences in bioavailability, metabolic pathways, or Omega-3 fatty acid needs among different populations. In summary, the findings of this study highlight the potential role of dietary Omega-3 intake in modulating PhenoAgeAccel and provide important evidence for further nutritional intervention research. However, more research is needed to explore the specific mechanisms by which Omega-3 intake influences aging and how these findings can be most effectively utilized to design health intervention strategies for diverse populations.

Recent research has unveiled the intricate interplay between Omega-3 fatty acids and aging, offering new insights into this relationship. Initially, Omega-3 fatty acids, particularly DHA and EPA, have been demonstrated to enhance brain function and structure in the elderly, exhibiting significant neuroprotective effects (23–25). Studies indicate that (9) Omega-3 fatty acids can increase levels of various signaling factors associated with synaptic plasticity, leading to an increase in dendritic spines and synapses, as well as enhanced neurogenesis in the hippocampus, even in old age. These effects are crucial for maintaining brain health and cognitive function, especially considering the increased risk of cognitive decline and Alzheimer’s disease in the elderly. Furthermore, the potential of Omega-3 fatty acids in anti-inflammatory action cannot be overlooked (26). A study utilizing multi-omics data unraveled the anti-aging mechanisms of Omega-3 fatty acids, finding that they can influence the methylation and expression levels of genes associated with age-related diseases or pathways. This finding further supports the possibility of Omega-3 fatty acids slowing the aging process through the regulation of gene expression. The impact of Omega-3 fatty acids on immune cells is also a significant aspect of their anti-aging action (27–29). These fatty acids can significantly influence the activation of the immune system, including regulating the properties of cell membranes and the assembly of complexes in lipid rafts, as well as functioning as signaling molecules. During aging, the decline in immune system function is a key factor leading to increased susceptibility to diseases, hence, by modulating immune responses, Omega-3 fatty acids may help slow this process. Lastly, the effect of Omega-3 fatty acids on mitochondrial function is key to their anti-aging action, with studies showing that Omega-3 fatty acids can improve mitochondrial function in the brains of aged mice, including increased ATP production and reduced oxidative stress (30). Given that mitochondrial dysfunction is associated with various age-related diseases, the potential of Omega-3 fatty acids to slow aging by improving mitochondrial function warrants further exploration.

Thus, the mechanisms by which Omega-3 fatty acids delay aging are complex and varied, affecting everything from the molecular level to the entire physiological system. At the molecular level, Omega-3 fatty acids, especially DHA and EPA, significantly influence the fluidity and signaling functions of cell membranes (31, 32). The presence of these fatty acids improves the structure and function of neuronal membranes, thereby affecting neurotransmitter release and neural signaling. Additionally, Omega-3 fatty acids, by modulating gene expression regulation, are involved in regulating anti-inflammatory and anti-oxidative stress responses, which are crucial for preventing age-related diseases such as cardiovascular and neurodegenerative diseases (33–35). In terms of cellular function, the protective role of Omega-3 fatty acids on mitochondria is undeniable. Mitochondria play a central role in cellular energy metabolism and the regulation of cell death, and Omega-3 fatty acids contribute to cellular health and function by maintaining the integrity of mitochondrial membranes and protecting mitochondria from damage (36). Furthermore, the regulatory effect of Omega-3 fatty acids on the immune system, through reducing the production of inflammatory mediators and modulating the activity of immune cells, is an important aspect of their aging-delaying action (37, 38). The above studies provide further support for the findings of this study, suggesting that Omega-3 fatty acids slow the aging process through various pathways, including improving brain function and structure, reducing inflammation, modulating immune responses, and improving mitochondrial function. The cumulative effect of these actions may provide a theoretical basis for developing anti-aging interventions based on Omega-3 fatty acids.

This study aimed to explore the relationship between dietary intake of Omega-3 fatty acids and accelerated phenotypic aging, enriching the theoretical foundation in the field of aging science and offering substantive guidance for public health practices. Utilizing large sample data from the NHANES database, this study revealed a significant negative correlation between Omega-3 fatty acid intake and PhenoAgeAccel, underscoring the importance of considering dietary factors in anti-aging strategies. Additionally, this research identified a nonlinear relationship and a threshold effect between Omega-3 intake and PhenoAgeAccel in a population for the first time, providing a new perspective for subsequent nutrition and aging studies.

However, as a scientific inquiry, this study inevitably has limitations. Firstly, due to its cross-sectional design, the study cannot establish a causal relationship between Omega-3 fatty acid intake and PhenoAgeAccel. Moreover, despite efforts to control for a wide range of potential covariates, the influence of all unmeasured variables cannot be entirely excluded. Furthermore, the use of traditional self-reported dietary assessment tools, such as food diaries or food frequency questionnaires, has significant limitations. These methods rely on participants’ memory and self-reporting, which are susceptible to recall bias and social desirability effects, leading to potential inaccuracies in reported intake. Therefore, it is recommended that future research incorporate quantitative nutrient biomarkers to more accurately measure blood levels of Omega-3 fatty acids, thereby enhancing the reliability and accuracy of study findings (39). Lastly, given the geographical and demographic characteristics of the study sample, the generalizability of its conclusions may be limited.

Nonetheless, the findings of this study lay a solid foundation for future research directions, especially in designing long-term prospective studies and randomized controlled trials to further explore the causal relationship between Omega-3 fatty acid intake and the aging process. Future research should also consider using more precise dietary assessment tools and validating the findings of this study in a broader population to enhance the external validity of the results.

In summary, this study provides valuable insights into the potential role of dietary Omega-3 fatty acids in modulating the human aging process, holding significant theoretical and practical implications for formulating evidence-based nutritional intervention strategies to delay aging and improve life quality. We look forward to subsequent research building upon this study to deepen the understanding of this field and offer more precise guidance for the health maintenance of the elderly population.

## 5 Conclusion

This study unveiled a significant negative correlation between dietary intake of Omega-3 fatty acids and PhenoAgeAccel, with the relationship stabilizing beyond an intake of 1.103 grams/day. The findings suggest that moderate increases in Omega-3 intake could have a positive effect on delaying aging, exhibiting certain variabilities across different populations. This study underscores the importance of considering Omega-3 fatty acid intake in public health practices and provides a theoretical foundation for future related research.

## Data Availability

The raw data supporting the conclusions of this article will be made available by the authors, without undue reservation.
